# Integration of in vitro and in-silico analysis of *Caulerpa racemosa* against antioxidant, antidiabetic, and anticancer activities

**DOI:** 10.1038/s41598-022-24021-y

**Published:** 2022-12-02

**Authors:** Indeewarie H. Dissanayake, Upeka Bandaranayake, Lakshika R. Keerthirathna, Chamalika. Manawadu, Rajitha M. Silva, Boudjelal Mohamed, Rizwan Ali, Dinithi C. Peiris

**Affiliations:** 1 Department of Zoology, University of Sri Jayewardenepura, Nugegoda, 10250 Sri Lanka; 2 Department of Statistics, University of Sri Jayewardenepura, Nugegoda, 10250 Sri Lanka; 3 Light and Electron Microscopy Unit, Medical Core Facility and Research Platforms, King Abdullah International Medical Research Centre, National Guard Health Affairs, Mail Code 1515, P.O. Box 3660, Riyadh, 11481 Saudi Arabia; 4 King Abdullah International Medical Research Center (KAIMRC), Medical Research Core Facility and Platforms (MRCFP), King Saud bin Abdulaziz University for Health Sciences (KSAU-HS), Ministry of National Guard Health Affairs (MNGHA), Riyadh, 11481 Saudi Arabia; 5 Department of Zoology/Genetics and Molecular Biology Unit (Centre for Biotechnology, University of Sri Jayewardenepura, Nugegoda, 10250 Sri Lanka

**Keywords:** Pharmacology, Pharmaceutics

## Abstract

Marine algae are found to be excellent in their nutritional and potential therapeutic properties. This study explores the antidiabetic and anticancer potential of fractionated polyphenolic extract of *Caulerpa racemosa,* green macroalgae. Crude polyphenolic extract (CPE) of *C. racemosa* and its fractions (n-hexane, ethyl acetate, chloroform, and distilled water) were tested for its total phenol and flavonoid contents and antioxidant potential. The ethyl acetate fraction was subjected to gas chromatography/mass spectrometry (GC/MS). The in vitro antidiabetic activity was assessed by alpha-amylase, glucosidase inhibition and anti-glycation assays. Also, in-silico studies were conducted to test the binding affinities between caulerpin with alpha-glucosidase enzyme and estrogen receptor (ER) active sites. Each fraction was tested for its in vitroin vitroanticancer activity by CellTiter-Glo and MTT cell proliferation assays. The total phenolic and flavonoid contents and the antioxidant potential of the crude extract were observed to be dose dependent. The GC/MS analysis of the ethyl acetate fraction yielded 47 peaks, whereas n-hexadecanoic acid and hexadecanoic acid methyl ester showed the highest compatibility percentages of 99% and 96%, respectively. The CPE exhibited a higher potential in both alpha-amylase inhibitory and anti-glycation activities. The ethyl acetate fraction was more effective against alpha-glucosidase inhibition. Molecular docking revealed a high binding affinity between the alpha-glucosidase enzyme and caulerpin and showed high binding affinity toward caulerpin, with H-bond interactions. The in vitro anticancer analyses revealed that chloroform fraction and CPE exhibited moderate activity on the KAIMRC1 cell line. Also, the CPE exhibited high specificity compared to the standard drug in anticancer studies. Our findings evidence the pharmacological potential of the CPE of *C. racemosa,* and bioactive compounds of the species may be utilized as lead molecules to develop anti-diabetic and anti-cancer drugs.

## Introduction

Current global statistics on non-communicable diseases reveal that cancer remains a growing health issue. To a certain extent, cancer is preventable by reducing the risk. Some preventive strategies include avoiding agents (biological, physical, and chemical) causing cancer and continual consumption of foods that exhibit cancer-protective effects. Although the current research trend focuses on synthetic chemotherapeutic drugs, conventional chemotherapy with synthetic drugs is proven to elicit severe complexities^1^.

Diabetes mellitus is characterized by alterations in carbohydrate, protein, and lipid metabolisms due to insulin secretory defects, insulin action or both. As per recent data, diabetes is on the rise from 143 million persons to 300 million by 2025^2^. As a treatment practice, postprandial hyperglycemia is reduced by lowering glucose absorption via inhibiting the carbohydrate hydrolyzing enzymes, such as amylase and glycosidase, in the digestive tract. These enzyme inhibitors also retard the time required for carbohydrate digestion, causing a reduction in the glucose absorption rate, which blunts the rise of the postprandial plasma glucose level^3^.

Unlike terrestrial organisms, marine organisms have not been widely employed in traditional medicine. But within the last 50 years, technological advances and innovative engineering have enabled the marine environment for scientific experiments^4^. Algae can be considered economically and ecologically impotent compared to other aquatic organisms. By producing various unique secondary (biologically active) metabolites, photosynthetic macro and microalgae adapt to withstand harsh environments^5^. Algae are excellent sources of nutrients such as protein, dietary fibre, fatty acids, vitamins, and macro and trace elements that have been reported to have both nutritional and functional properties and their potential use as therapeutic agents^6,7^. As a result, there is an emerging interest in promoting the health status of humans and other animals using marine-derived natural resources^8^. The genus *Caulerpa* consists of *Caulerpa racemosa* (Forsskal) J. Agardh belonging to the order Bryopsidales, family Caulerpaceae, which includes approximately 85 species^9^. A complex of *C. racemosa* contains spherical, club-shaped, or mushroom- to disc-shaped branchlets with erect fronds (usually up to 11 cm high) that bear radially or distichously arranged vesiculate ramuli. Therefore, it can be distinguished from the feather-like flat *Caulerpa taxifolia* species^10^.

The proximate chemical composition analyses of *C. racemosa* collected from St. Martin Island, Bangladesh, was found to contain a higher amount of carbohydrates (48.97 ± 1.22%), protein (19.72 ± 0.77%), crude lipid (7.65 ± 1.19%) and fibre (11.51 ± 1.32%) contents. Also, an ash content of 12.15 ± 0.46% and moisture content of 15.37 ± 0.72% were recorded^11^. The study further revealed that glutamic acids (9.2 ± 0.7%) and aspartic acids (12.7 ± 0.2%) were the most abundant amino acid in *C. racemosa* with a higher percentage of isoleucine (5.8 ± 0.3%) and threonine (6.2 ± 0.5%) and lower percentages of leucine (6.9 ± 0.6%) and valine (5.1 ± 0.3%) of the total amino acid were also recorded. Another chemical composition analysis of polysaccharides from *C. racemosa* collected from the South China Sea, Zhanjiang, China, revealed a higher percentage of total sugars (53.7%), Sulphate groups (27.6%), proteins (9.9%), and Uronic acid (7.9%), revealing that all polysaccharides are proteoglycans rich in sulphate groups^12^. The authors also reported that the primary amino acids contained in *C. racemosa* are aspartic and glutamic acids, followed by other amino acids such as alanine.^13^.

An untargeted metabolomic profiling test of sea grape *C. racemosa* using the liquid chromatography–high-resolution mass spectrometry (LC-HRMS) revealed six major compounds with mzCloud MS/MS library values > 75%, including 2-(1H-indol-3-yl)-3-[4- (trifluoromethyl)phenyl]acrylonitrile (ITPA), Choline and Betaine.

However, due to the uniqueness of each alga with its biochemical characteristics, there is an urgent need to explore its multifunctional properties at their maximum level. The present study intends to investigate the in vitro antioxidant, antidiabetic, and anticancer (against human carcinoma cell lines) activities of fractionated polyphenolic extract of *Caulerpa racemosa*. We further extended the study to determine the drug-like behaviour of the established isolated compound caulerpin for cancer and diabetes.

## Results

### Total phenol and flavonoid contents

A dose-dependent total phenol and flavonoid contents were observed. The entire phenol content ranged from 0.010 to 0.013 (GAE/g) of dry mass for the concentrations used. Likewise, dry mass of total flavonoid content ranged between 0.001 to 0.003 (QC/g). It was evident that the total phenolic content was considerably higher (a fewfold) at a given concentration than the total flavonoid content (Table 1). However, a positive correlation between total flavonoids and phenolics was observed at increasing concentrations, with decreasing fold values.

**Table 1:** Total phenol (TPC) and flavonoid contents (TFC) of the CPE (crude polyphenolic extract).

Concentration (mg/ml)	TPC (GAE/g)	TFC (QC/g)
0.03125	0.01054 ± 0.008	0.0010 ± 0.003
0.0625	0.01138 ± 0.006	0.0013 ± 0.0005
0.125	0.01180 ± 0.0102	0.00155 ± 0.007
0.25	0.0120 ± 0.004	0.00176 ± 0.001
0.5	0.0128 ± 0.003	0.00228 ± 0.0005

Values are presented as the mean ± standard error mean (*n* = 3).

**Figure 1:**
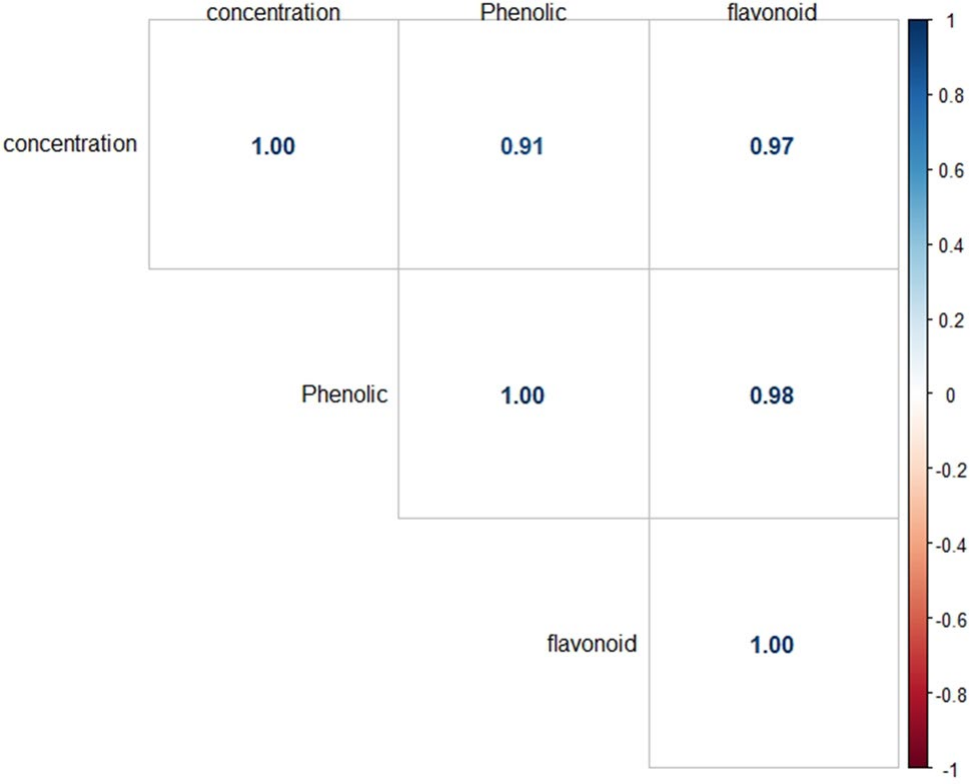
Correlation matrix showing the concentration of the crude polyphenolic extract (CPE) with total phenolic and flavonoid contents.

**Figure 2:**
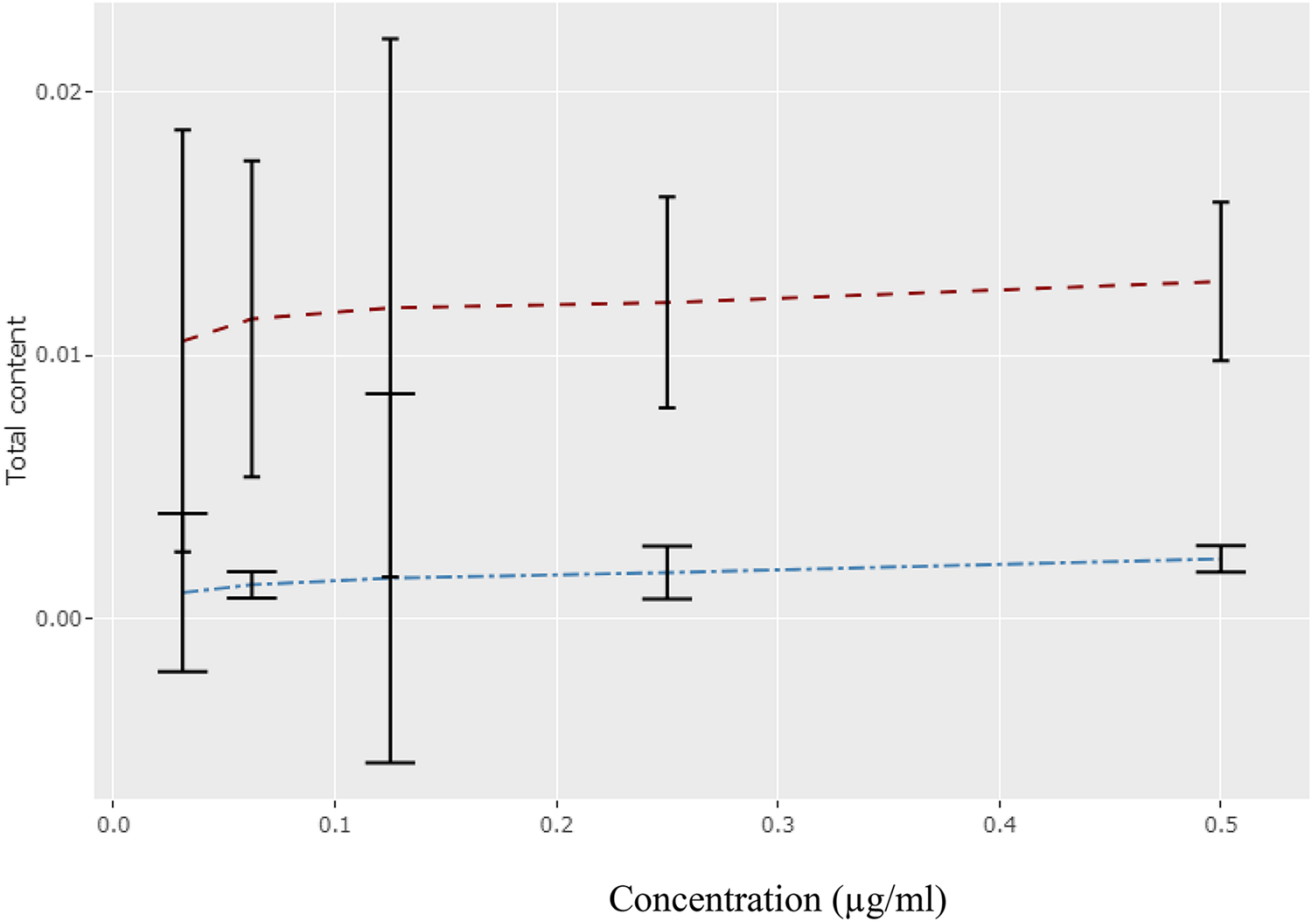
Correlation plot showing the concentration of the crude polyphenolic extract and total phenolic and total flavonoid contents.

A correlation between the concentration of the CPE with total phenolic and total flavonoid contents was analyzed in the correlation matrix (Fig. 1). A strong correlation is indicated between engagement and phenolic and flavonoid content, with 91% and 97%, respectively. It also shows a strong correlation between phenolic and flavonoid contents of 98%. The correlation between phenolic and flavonoid contents is further described in Fig. 2.

### Antioxidant activity

**Figure 3:**
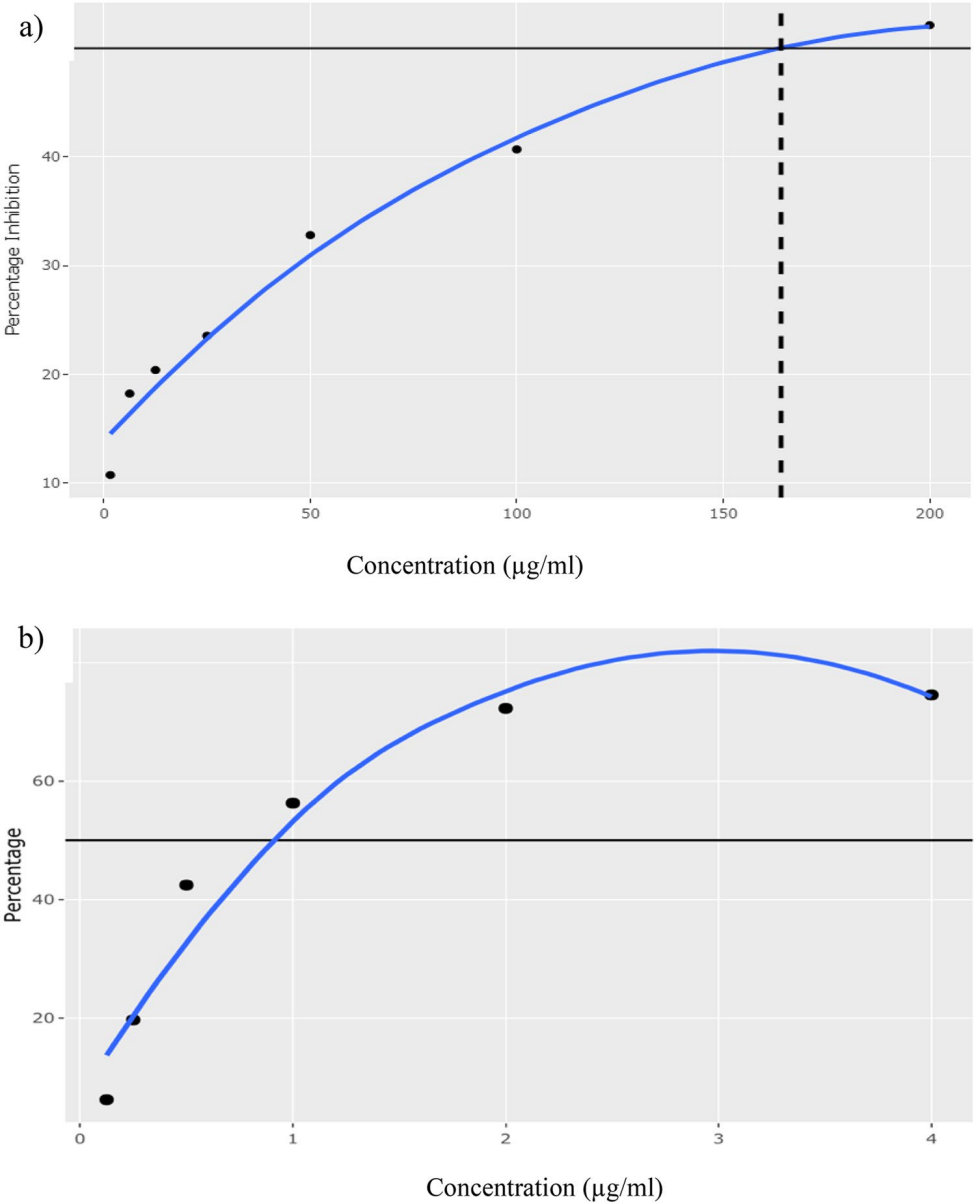
Percentage inhibition exhibited by (**a**) crude polyphenolic extract and (**b**) ascorbic acid against DPPH radical scavenging.

The act of free radical scavenging defines antioxidative properties due to redox properties. The antioxidative property is essential for alleviating numerous ailments, including cancer, as it reduces oxidative stress. The antioxidant potential was evaluated using DPPH (2, 2-diphenyl-1-picryl-hydrazylhydrate) radical photometric assay, a process guided by its discolouration. A dose-dependent antioxidant activity was observed with the concentrations used. The CPE showed an IC_50_ value of 164.83 µg/ml compared to the positive control-Ascorbic acid, which showed an IC_50_ value of 49.88 µg/ml (Fig. 3).

**Figure 4:**
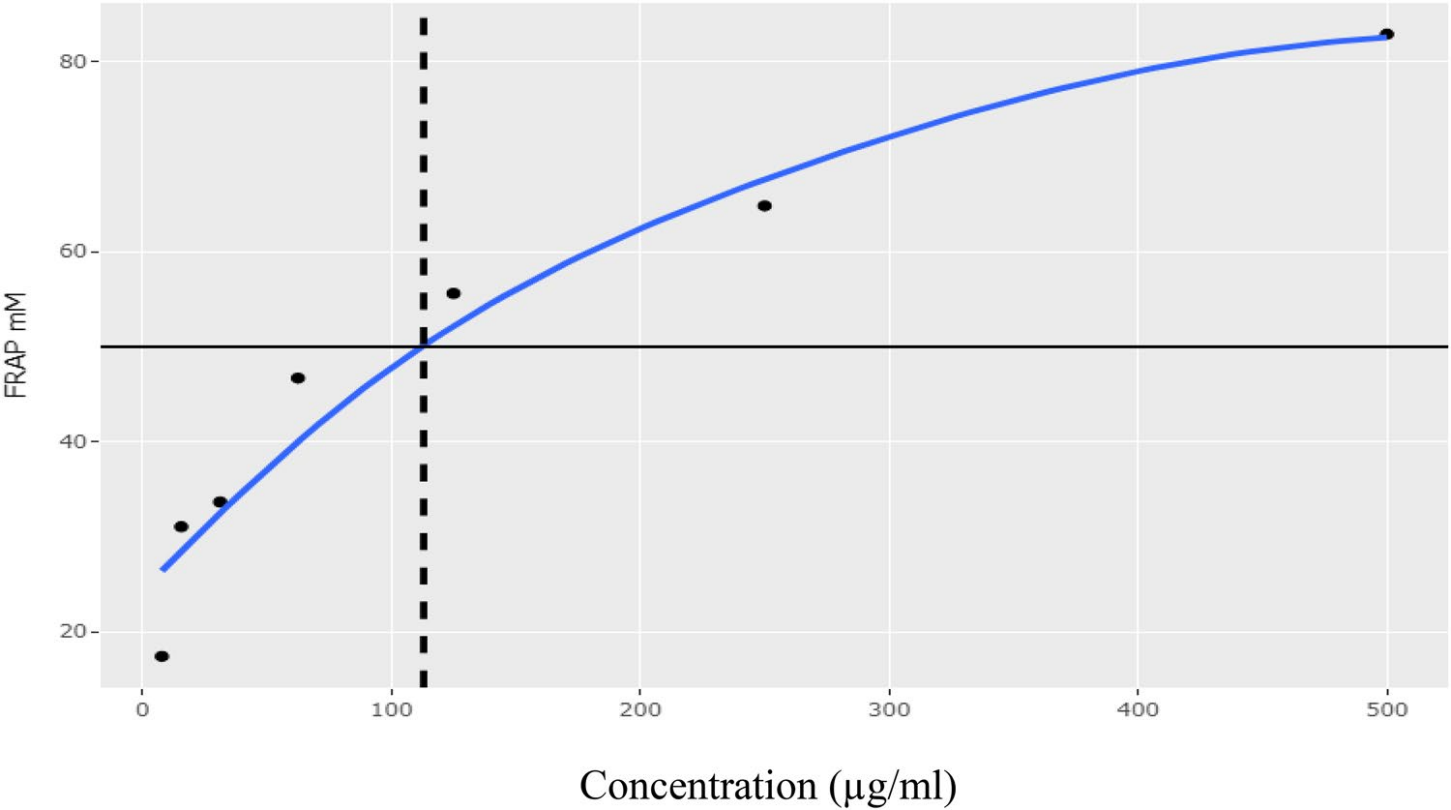
Percentage inhibition of FRAP assay of the crude polyphenolic extract.

The principle of FRAP assay is based on the ability of pH to reduce Fe^3+^ to Fe^2+.^ The values were obtained by comparing the absorbance change in the test mixture obtained from increasing concentrations of Fe^3+^ and expressed as mg of Trolox equivalent/g. According to Fig. 4, *C. racemosa* showed increased ferric reducing power with increasing concentrations with an (IC_50_ value of 113.73 µg/ml.

### Gas chromatography/mass spectrometry (GC/MS) analysis

Since the ethyl acetate fraction of *C. racemosa* gave promising results (Table 2), it was subjected to GC–MS analysis. The ethyl acetate fraction yielded 47 peaks, while n-hexadecanoic acid and hexadecanoic acid methyl ester showed the highest compatibility percentages of 99% and 96%, respectively.

**Table 2:** Compounds available in ethyl acetate fraction of *Caulerpa racemosa.*

Peak no	Retention time (min)	Compound	Molecular formula	MW (g/mol)	Peak area %
1	3.258	Acetaldehyde, O-ethyloxime	C_3_H_7_NO	73.09	63.869
2	4.109	Propanoic acid, ethyl ester	C_5_H_10_O_2_	102.13	7.500
3	4.292	Formic acid, butyl ester	C_5_H_10_O_2_	102.13	0.828
4	4.708	Acetic acid, butyl ester	C_6_H_12_O_2_	116.16	0.642
5	5.141	Pentane, 1-butoxy	C_9_H_20_O	144.25	0.075
6	5.273	n-Propyl acetate	C_5_H_10_O_2_	102.131	0.154
7	6.469	1-Butanol	C_4_H_10_O	74.121	3.919
8	6.769	Acetic acid	CH_3_COOH	60.052	22.514
9	15.805	2,5-Hexanediol, 2,5-dimethyl	C_8_H_18_O_2_	146.13	1.375
10	16.194	4-Hydroxypyridine 1-oxide	C_5_H_5_NO_2_	111.10	0.084
11	16.940	Cyclobutane, 1,2-diethyl	C_8_H_16_	112.21	0.056
12	20.235	Tricosyl trifluoroacetate	C_25_H_47_F_3_O_2_	436.6	0.116
13	21.533	2-Butanone, 4-phenyl	C_10_H_12_O	148.20	0.067
14	24.217	1-Hexadecene	C_16_H_32_	224.42	0.074
15	25.671	Hexadecane	C_16_H_34_	226.41	0.056
16	28.417	Acetic acid, 2-(pyridin-2-ylamino) cyclohexyl ester	C_13_H_18_N_2_O_2_	234.29	0.362
17	28.534	Phosphorodithioic acid, O-ethyl S,S-diphenyl ester	C_14_H_15_O_2_PS_2_	310.4	0.484
18	28.783	5-Bromo-2-methoxy-2,4,6-cycloheptatrien-1-one	C_8_H_7_BrO_2_	215.04	0.149
19	28.858	Spiro(cyclohexane-1,2'-(1,3-dithiolane))	C_8_H_14_S_2_	174.33	0.006
20	28.879	( +)-3-Carene, 2- alpha-isopropenyl	C_13_H_20_	176.30	0.003
21	31.569	6H,8H-Isoquino[2,1-c][1,3] benzoxazine, 9,13b-dihydro-11,12-dimethoxy-6-methyl	–	–	0.150
22	31.742	2-Butanone, 4-(4-methoxyphenyl)	C_11_H_14_O_2_	178.23	0.146
23	31.924	Hexadecenoic acid, methyl ester	C_17_H_34_O_2_	270.45	0.150
24	32.847	1,4-Dioxane, 2,3-dichloro	C_4_H_6_Cl_2_O_2_	156.99	0.215
25	32.943	N-Ethyl-4-nitroaniline	C_8_H_10_N_2_O_2_	166.18	0.139
26	33.818	Benzoic acid, 4-[3-(5-cyclopropyl-2H-pyrazol-3-yl)-5-mercapto-[1,2,4]triazol-4-yl]	–	–	0.347
27	34.006	5.alpha.,14.beta.-Spirostan-3-one, cyclic ethylene acetal, (25R)	–	–	0.071
28	34.656	1H-Pyrido [3,4-bindole, 2,3,4,9-tetrahydro-6-methoxy-1-methyl	C_13_H_16_N_2_O	216.28	0.716
29	35.221	[1,2,4]Oxadiazole, 3-(5-bromofuran-2-yl)-5-(4-methoxybenzyl)	–	–	0.525
30	35.554	3H-1,2,4-Triazole-3-thione, 2,4-dihydro-4-phenyl	C_14_H_12_N_4_S	268.34	0.376
31	36.040	Benzenehexanamine	C_12_H_19_N	177.29	6.693
32	36.540	2H-Pyrimido[2,1-b][1,3]thiazin-6-one, 8-methyl-3,4-dihydro	C_8_H_10_N_2_O_S_	182.24	0.757
33	36.599	Gona-1,3,5,7,9-pentaen-17-one, 13-ethyl-3-hydroxy	C_19_H_20_O_2_	280.4	0.625
34	37.115	9H-Benzo[4,5]imidazo[2,1-c][1,2,4]triazole, 3-benzylsulfanyl	C_15_H_12_N_4_S	280.35	3.008
35	37.269	n-Hexadecanoic acid	C_16_H_32_O_2_	256.4	5.742
36	37.703	Nicotinonitrile, 2-[2-(4-bromophenyl)-2-oxoethoxy]-4-methoxymethyl-6-methyl	–	–	2.449
37	38.074	3-Phenyl-7-[2,4-bis(diethylamino)-1,3,5-triazin-6-yl]aminocoumarin	–	–	0.995
38	38.621	5-Fluoro-1,3-bis[phenylmethyl]-2,4(1H,3H)-pyrimidinedione	C_18_H_15_FN_2_O_2_	310.3	4.850
39	38.869	Xanthine, 2,4,7-trimethyl-8-[2-[2-methylphenyl]ethenyl]	–	–	0.733
40	39.459	4,4'-Bis[2-hydroxyhexafluoroisopropyl]diphenyl ether	C_18_H_10_F_12_O_3_	502.2	4.256
41	39.767	5-[4-Methoxyphenoxy]-6-amino-8-methoxyquinaldine	–	–	3.770
42	40.370	4-Nitro-4'-sulfamyldiphenyl sulfide	–	–	6.376
43	41.207	8-Naphthol, 1-(benzyloxy)	C_17_H_14_O_2_	250.29	11.275
44	42.535	Tetradecanoic acid, 2-hydroxy-, monoanhydride	C_14_H_28_O_3_	244.3	29.354
45	43.561	Estra-1,3,5(10)-trien-17-one, 3,12-bis[(trimethylsilyl)oxy]-, O-methyloxime, (12.beta.)	–	–	19.495
46	43.715	2-(p-Anisidino)-4-methyl-8-nitroquinoline	–	–	20.871
47	46.339	No hits found	–	–	1.321

### Anti-diabetic activity

**Figure 5:**
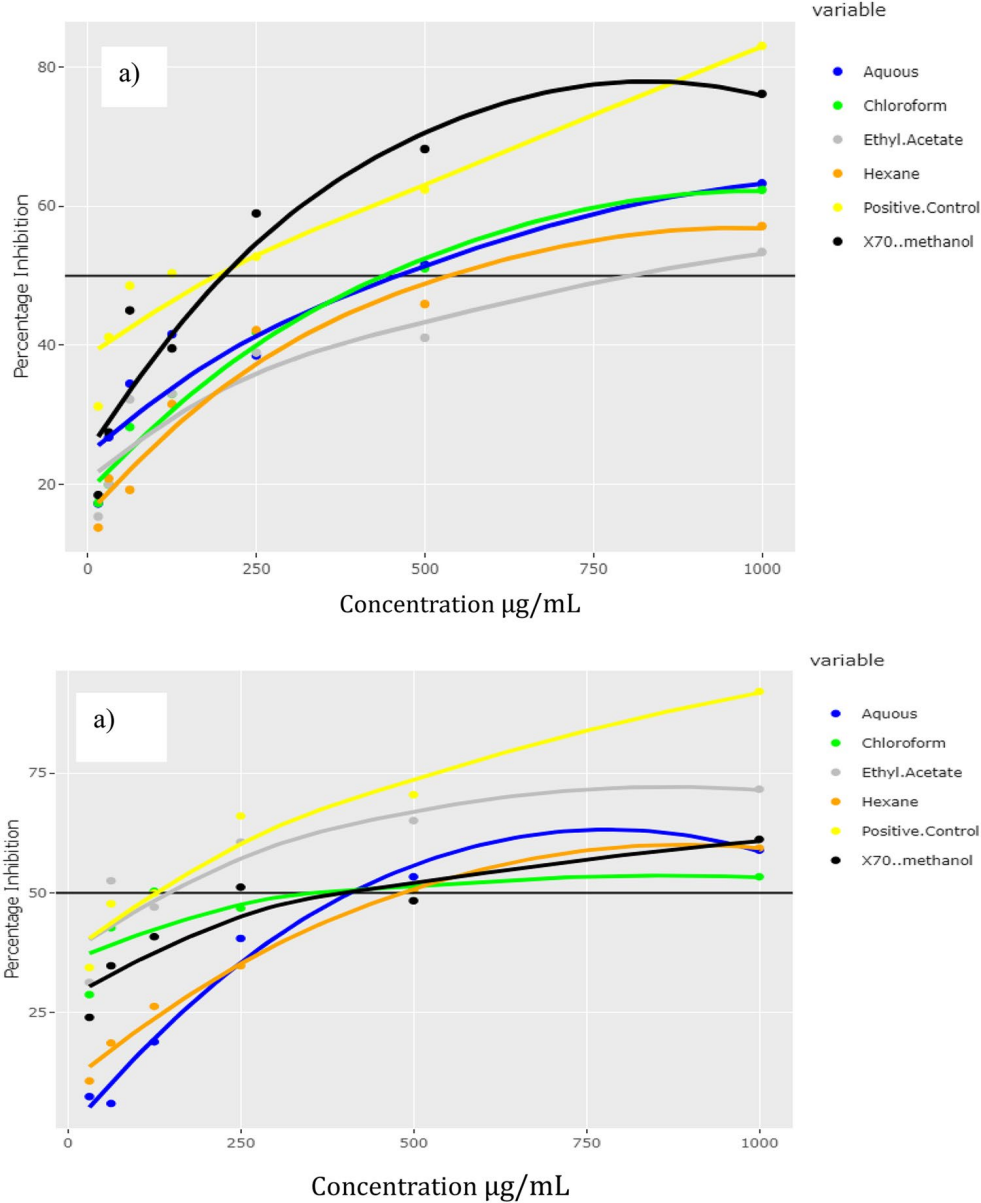
Percentage of alpha-amylase (**a**) and alpha-glucosidase (**b**) inhibitory activities exhibited by the standard drug (metformin), the crude polyphenolic extract and different fractions of *Caulerpa racemosa.*

The percentage inhibitory activities exhibited by the CPE and its fractions are displayed in Fig. 5. According to the results, the samples exhibited a dose–response relationship. When comparing the IC_50_ values, the crude polyphenolic extract exhibited the highest inhibitory activity of α-amylase with an IC_50_ value of 202.53 µg/ml, which was equal to the positive control. The lowest IC_50_ value was exhibited by the ethyl acetate fraction. The inhibition of alpha-glucosidase increased in the order of hexane < aqueous < CPE < chloroform < ethyl acetate fraction. The ethyl acetate fraction exhibited a potent α-glucosidase inhibitory activity (IC_50_: 153.87 µg/ml) and was comparable to the standard drug (IC_50_: 125.00 µg/ml) and other fractions (Table 3).

**Table 3:** The IC_50_ (μg/ml) values of α-amylase and α-glucosidase enzymes inhibitory activities of the crude methanol extract of *C. racemosa*, its fractions (chloroform, hexane, ethyl acetate, and aqueous), and the standard drug.

Extract/fraction	Alpha-amylase		Alpha-glucosidase
	IC_50_ (µg/ml)		
Crude polyphenolic extract	202.53 ± 2.43	399.13 ± 0.89	
Hexane	538.96 ± 3.73	484.96 ± 4.23	
Ethyl acetate	800.63 ± 4.70	153.87 ± 2.37	
Aqueous	464.20 ± 5.62	411.39 ± 1.62	
Chloroform	451.74 ± 2.14	362.34 ± 1.87	
Positive control (acarbose)	190.07 ± 2.42	125.00 ± 1.06	

### Antiproliferative activity

When considering the potency of proliferation inhibition of the crude polyphenolic extract and its fractions, the CPE, chloroform, and aqueous fractions showed some potential for breast cancer cell lines. For example, chloroform (IC_50_: 92.15 µg/ml) and the CPE (IC_50_: 168.5 µg/ml) exhibited more potent activities on the KAIMRC1 cell line, whereas the aqueous extract (IC_50_: 48.31 µg/ml) showed high activity on MCF-7 cell line. Similarly, the CPE showed high activity in the colorectal HCT-8 cell line (IC_50_: 160.0 µg/ml). The results are depicted in Table 4.

**Table 4:** IC_50_ values (µM) exhibited by the crude polyphenolic extract (CPE) and its fractions (**H**: hexane, C: chloroform, EA: ethyl acetate, Me: methanol; Aq: aqueous) of C. *racemose* and the standard drug (M: mitoxantrone) on different cancer (breast, colorectal, hepatoma and leukemia and normal (epithelial and monocular blood) cell lines.

	Sample	Breast cancer cell lines			Colorectal	Hepatoma	Leukemia			Control cell lines	
		KAIMR C1	MDA-MB-231	MCF-7	HCT-8	Hep G2	KG-1a	K-562	HL-60	Human epithelial	PBMC
IC _50_ values (µM)	CPE	**168.5**	304.1	–	**160.0**	1570	644.7	2768	914.7	756.6	2940
	H										
	C	**92.91**	3046	546.8	2344	3848	1388	3001	655.6	7412	1102
	EA	1722									
	Me	1194									
	Aq	515	**118.0**	**48.31**							
	M	0.638	0.520	1.224	0.164	0.140	0.145	0.585	–	0.12	0.142

Significant values are in bold.

### Selectivity indices

Among the crude extract, fractions, and the standard drug, only the CPE, chloroform fraction, and the standard drug exhibited inhibitory activities against normal epithelial and blood cell lines. According to the selectivity index, a value > 1 indicates a considerable anticancer specificity, whereas a value much larger than 1 indicates very high selectivity (Table 5).

**Table 5:** Selectivity indices of the crude polyphenolic extract, the chloroform fraction, and the standard drug (mitoxantrone).

Sample	Breast cancer cells		Colorectal cancer cells	Leukaemia		
	KAIMRC1	MD-AB-231		KG-1a	K-562	HL-60
Crude polyphenolic extract	4.49	2.48	4.73	4.56	1.06	3.21
Chloroform fraction	− 79.21	− 2.4	− 3.16	0.79	0.37	1.68
Mitoxantrone	0.19	0.23	0.86	0.98	0.24	–

The specificity of the CPE and its fraction increased in the order of CPE-K562 (1.06) < chloroform fraction-HL-60 (1.68) < CPE- MD-AB-231 (2.48) < CPE- HL-60 (3.2i1) < CPE-KAIMRC1 (4.49) < CPE-KG1a (4.56), and CPE-colorectal cancer cells (4.73). The standard drug mitoxantrone did not exhibit cell specificity, indicating that it affects healthy cells. The highest specificity was observed with the CPE against colorectal cancer, breast cancer (KAIMRCI), and leukemia (KG-1a), indicating the potential benefit of the CPE against the said cancers.

### In silico analysis

Caulerpin belonging to a bisindole alkaloid group with the chemical structure of dimethyl 5,12-dihydrocycloocta [1,2-b:5,6-b'] diindole-6,13-dicarboxylate (molecular formula: C_24_H_18_N_2_O_4_).

Caulerpin was subjected to docking studies against α-glucosidase and acarbose (standard drug) using the Auto dock tools suite, and the results are illustrated in Fig. 7A,B. Also, we conducted a docking analysis to study the binding affinities of caulerpin to estrogen receptor (ER), and the FDA-approved drug mitoxantrone.

**Figure 6:**
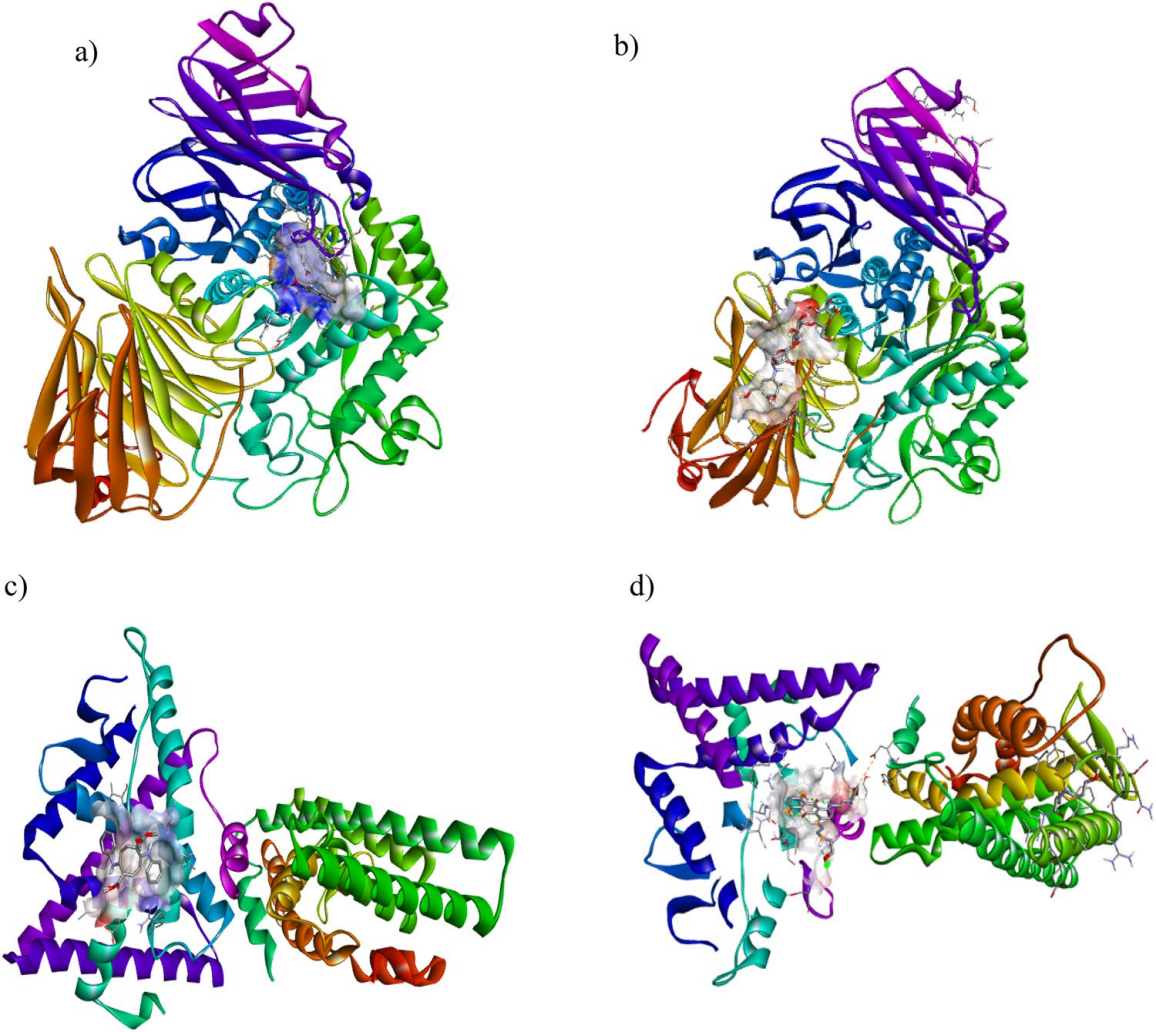
The three-dimensional surface structure-forming complexes between the alpha-glucosidase (**a**) and acarbose (**b**; standard drug) with caulerpin. (**c**) Estrogen receptor alpha and caulerpin; (**d**) mitoxantrone (FDA approved drug) caulerpin complexe.

**Figure 7:**
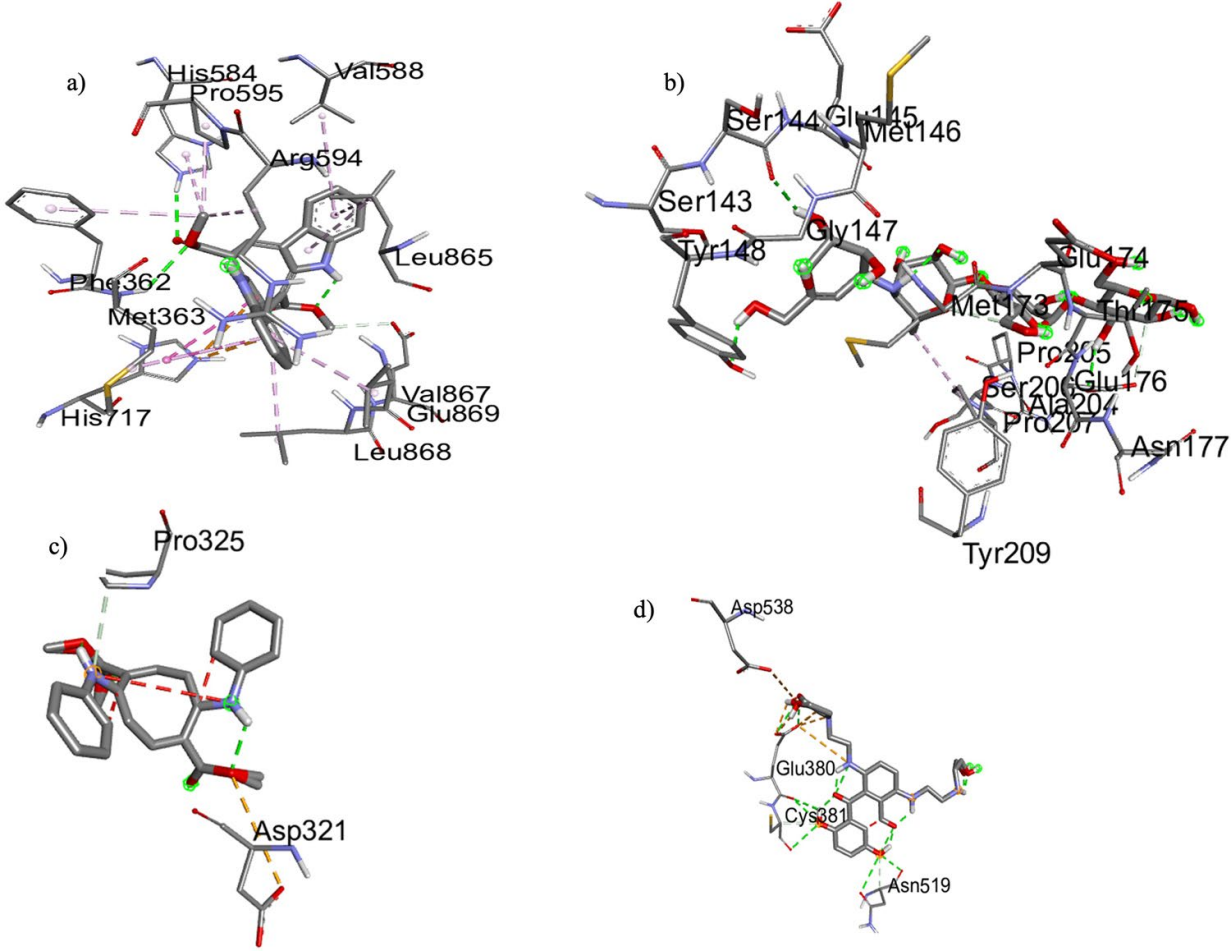
Binding posepre dictions for docked ligands (green) superposed on crystallographic structures for six representative targets. Key hydrogen bonds are shown by yellow lines, and the protein molecular surface is colored by atom type. Images generated with Chimera.

The resulting docking log file cluster analysis revealed that the best-docked conformation of the ligand and human pancreatic α-glucosidase protein docking studies with the best-selected pose had binding energy of − 6.13 kcal/mol, and the best-docked conformation is shown in Fig. 6a. The 2D analysis of the active site showed the involvement of van der Waal forces between the ligand and three residues from the protein, HIS584 and MET363 (Fig. 7a). Similarly, the best-docked conformation of alpha-glucosidase protein with acarbose was cluster 10, which had binding energy of + 0.75 kcal/mol (Fig. 6b) and formed three hydrogen bonds with SER144, TYR148 and GLU176 (Fig. 7b). The docking energy was lower in the novel ligand in comparison to the approved drug acarbose. The number of hydrogen bonds formed with acarbose is higher, which could help form a stable ligand-receptor complex.

The best-docked conformation between ER protein with novel ligand caulerpin was cluster 3, which had binding energy of − 11.18 kcal/mol (Fig. 6c), and no hydrogen bonds were found. Attractive charges and van der Waal bonds were formed between ASP231 and PRO325, respectively (Fig. 7c). Even though no hydrogen bond formation was observed, the lower binding energy could signify the potential of the ligand as a promising drug candidate. The best-docked conformation between ER protein and FDA-approved drug mitoxantrone was cluster 4, with a binding energy of − 8.45 kcal/mol (Fig. 6d). The drug formed three hydrogen bonds with residues GLU380, CYS381 and ASN519 (Fig. 7d). In comparing the two drug compounds, the mitoxantrone could potentially result in a more stable ligand-receptor complex due to the higher number of hydrogen bonds formed. Still, the lower overall binding energy in caulerpin is also noteworthy in assessing its feasibility as a drug compound.

## Discussion

The current study intended to decipher the in vitro antioxidant, antidiabetic, and anticancer activities of fractionated polyphenolic extract of *Caulerpa racemosa*, a marine alga. Further, we conducted ligand screening to study the drug-like behaviour of polyphenolic extract. Due to numerous health benefits, consuming food rich in antioxidants has become an essential part of the human diet^9^. The presence of phenols and flavonoids in natural food can be attributed to antioxidant capability^10^*. C. racemosa* is an alga rich in phenolic and flavonoids and widely consumed by humans. In this study, phenolics and flavonoids were quantified, and their correlation was studied. A strong correlation was obtained between total phenolic (TPC) and flavonoid content in the crude polyphenolic extract (CPE) of *C. racemosa* (98%), suggesting an abundance of phytochemicals with antioxidant potential. However, according to the IC_50_ values, the CPE showed moderately high antioxidant activity in both assays (DPPH IC_50_: 164.83 µg/ml and FRAP IC_50_: 113.73 µg/ml). Previously published literature^10,14^ confirmed the present findings, and it was stated that the moderate scavenging activity, lower reducing power and the chelating ability of *C. racemosa.* However, these studies have shown a positive correlation between TPC and FRAP in *C. racemosa*. Different results obtained for the correlations of the phytochemicals and two antioxidant assays can be attributed to the presence of ascorbic acid, folic acid, and thiamine in the radical assay and the ability of *C. racemosa* to scavenge them effectively.

Recent studies^12,15^ investigated the antioxidant activity of *C. racemosa* in its various extracts (crude ethanol, crude polysaccharides, methanol, chloroform, hexane), implementing several extraction techniques (microwave-assisted extraction, shaking and Soxhlet extraction). Their findings suggest that mainly phenolic compounds of medium polarity, degree of sulfation in fucoidans, and metabolomic adaptations due to the geographical variations contributed to the observed antioxidant activity. Fernando et al.^12^ studied isolated squalene of *C. racemosa* for the first time and identified its potent anti-inflammatory and antioxidant activities. However, squalene has not exhibited DPPH radical scavenging potential in the studied concentration range.

Results of the GC/MS analysis of the ethyl acetate fraction portray that *C. racemosa* is a rich source of potent bioactive compounds such as formic acid, butyl ester, acetic acid, butyl ester, 2-Butanone, 4-phenyl (benzyl acetone), hexadecane hexadecanoic acid, methyl ester, 1H-Pyrido[3,4-b] indole, 2,3,4,9-tetrahydro-6-methoxy-1-methyl and n-hexadecanoic acid. These compounds have demonstrated various biological activities, including antimicrobial, antioxidant, anti-inflammatory, anti-androgenic, hemolytic, and antipsychotic effects^15^. Previous studies^16,17^ conducted by many researchers have also shown that chloroform and methanol extracts of *C. racemosa* contained numerous bioactive compounds with the properties above metabolic profiling of *C. racemosa* revealed secondary metabolites, mainly flavonoids, in the *Caulerpa* species.

The evidence of hyperglycemia in either postprandial or fasting states is referred to as diabetes, and its overall prevalence is estimated to rise^18^. The body’s glucose homeostasis is altered either because of insulin resistance or impaired beta-cell function. Postprandial hyperglycemia in type 2 diabetes mellitus patients can be controlled by inhibiting metabolic carbohydrate digesting enzymes such as alpha-amylase, alpha-glucosidase, and dipeptide peptidase IV^19^. Therefore, inhibiting key metabolic carbohydrate-digesting enzymes is one of the main strategies to evaluate antidiabetic activity. Glycation end products are believed to perform a causative role in the vascular complications of diabetes. Glycation is also a biomarker for diabetes and is implicated in some diseases and ageing^20^. The current study employed three in vitro assays in determining anti-diabetic activity, demonstrating that the CPE (alpha-amylase IC_50_: 202.53 µg/ml) and ethyl acetate (alpha-glucosidase IC_50_: 153.87 µg/ml) as potent fractions.

A recent review conducted by a group of researchers stated that a species in the Caulerpa family- *Caulerpa lentillifera* contains polyphenols and sterols that decrease the level of dipeptide- peptidase IV and alpha-glucosidase enzymes. Furthermore, the species increases insulin secretion and glucose uptake in the 3T31 adipocytes^21^. The following section specifies a few studies that reported the in vitro and in vivo antidiabetic activity of *C. racemosa*. In vitro studies examined the alpha-amylase inhibitory activity of *C. racemosa* in ethyl acetate, methanol, and acetone extracts. The findings indicated a moderate alpha-amylase inhibition in the methanol extract^22^. This finding is consistent with our results as the crude polyphenolic extract is majorly methanol-extracted. Another in vitro approach examined the alpha-amylase and alpha-glucosidase inhibitory effects of *C. racemosa* in methanol, ethyl acetate, and hexane extracts^23^. The findings do not portray a considerable enzyme inhibitory effect compared to the other seaweeds used in the study. However, the ethyl acetate extract of *C. racemosa* displayed the highest alpha-amylase inhibition and moderate antioxidant activities compared to its ethanol counterparts.

An *in-vivo* approach examined the ethanolic extract of *C. racemosa* using various biochemical paradigms against glipizide (5 mg/kg) in a streptozotocin-induced diabetes rat model and reported significant outcomes^24^. The ethanol extract of *C. racemosa* remarkably reduced blood glucose. Also, the ethanol extract restored the impaired glycosylated hemoglobin level, glucose uptake by hemidiaphragm, liver glycogen level and glucose transport by hepatic cells. Moreover, a higher restoration effect was displayed in lipid abnormalities, elevated liver enzymes, elevated inflammatory markers, and depleted endogenous antioxidants in the pre-treatment with ethanolic extract of *C. racemosa*. Furthermore, the authors reported a safe and no effect from the ethanolic extract of *C. racemosa* on vital organs. Another recent in vivo study^15^, examined oral supplementation effects with whole powdered *C. racemosa* in a rat model induced type 2 diabetes. Oral supplementation effectively prevented liver lipid peroxidation and alleviated liver, renal and pancreatic tissue damages. Besides, Aroyehun et al.^25^ reported the efficacy of the ethyl acetate fraction of *C. racemosa* in diabetic-induced rats. The low (100 mg/kg) and high (200 mg/kg) doses of *C. racemosa* demonstrated notable anti-diabetic activity with a significant decrease (in blood glucose levels while preventing weight loss, reducing plasma ALT and AST levels as an indication of hepatoprotective effect in diabetic induced rats. Outcomes of these in vitro and in vivo studies show the promising antidiabetic activity of marine algae- *C.racemosa*, confirming the results obtained in the present study.

We further conducted molecular docking to predict the binding affinity of caulerpin (dimethyl 5,12-dihydrocycloocta [1,2-b:5,6-b'] diindole-6,13-dicarboxylate) or acarbose (standard drug), to the active site of α-glucosidase. The results revealed that caulerpin forms strong hydron bonds with HIS584 and MET363 and van der Waal interactions with seven amino acid residues. The standard drug formed three H-bonds with three hydrogen bonds SER144, TYR148 and GLU176.) and van der Waal forces with one amino acid. The results confirmed the potent in vitro antidiabetic activity exhibited by the ethyl acetate fraction of *C. racemosa* and were comparable to the standard drug. Further, comparing the free binding energy of best-docked confirmation of alpha-glucosidase enzyme and caulerpin (− 6.13 kcal/mol) and acarbose (+ 0.75 kcal/mol), the binding energy of caulerpin was nine-fold lower, indicating strong binding to the studied ligand. In a docking study by Rehman et al.^26^, *Saccharomyces cerevisiae* α-glucosidase enzyme and *C. racemosa* exhibited higher binding energy than in the present study. The increased binding capacity observed in the present study may be due to different sources of α-glucosidase enzyme employed for docking. Therefore, the CPE could assist medicinal chemists in designing inhibitors against alpha-glucosidase enzymes in the human intestine to overcome diabetic conditions.

A significant rise in cancer and its adverse reactions to synthetic drugs has facilitated considerable research in naturally derived therapeutic alternatives. Hence, the current study evaluated the anti-proliferative efficacy of fractionated polyphenols of *C. racemosa* against breast (KAIMRC1, MDAMB-23, and MCF-7), colorectal (HCT8), hepatoma (Hep G2), leukemia (KG1a, K562, and HL60) cancer cell lines and normal primary fibroblasts (P1) and blood sample (N1 and N2) cell lines. The current study revealed that fractions of chloroform, aqueous, and CPE (crude polyphenolic extract) have the potential for breast carcinoma cell lines. The chloroform fraction and the CPE exhibited moderate activity on the KAIMRC1 cell line (IC_50_: 92.91 and 168.5 µM), whereas the aqueous fraction showed moderate activity on the MCF-7 (IC_50_: 48.31 µM) and MDA-MB-231 (IC_50_:118.0 µM) cell lines. The. CPE also showed moderate activity on the colorectal HCT8 (IC_50_:160.0 µM) cell line. These results can be supported by previous studies evaluating the anti-cancer efficacy of the *C. racemosa* or *Caulerpa* genus that contained numerous putative anti-cancer effective compounds^27,28^.

Moreover, the CPE exhibited high specificity against leukemia, breast and colorectal cancer cell lines compared to the standard mitoxantrone drug. Typically, a decent anticancer drug should have a relatively high toxic concentration with high selectivity. A low selectivity value (< 1) indicates that a drug could harm non-malignant cells^29^. In the present study, the CPE exhibited high specificity implying its potential to develop as a novel cancer drug lead.

Recent literature revealed that *C. racemosa* exhibited a higher potential with a lower dose of the extract against the HeLa (human cervical) and the Huh-7 (human hepatoma) cancer cells) with observed upregulated transcript expression of the *p53* gene in the HeLa cells^30^. Lakmal et al.^31^ showed a distinct inhibitory effect of cancer cell growth (IC_50_ value 30.17 µM) in *C. racemosa* methanol extract against HL-60 cells. There have been few records of *C. taxifolia* exerting the anticancer potential against breast (MDA-MB-231, T-47D) and lung (H1299) cancer cells, together with plausible effects on mitochondrial membrane potential (MMP) and cell cycle progression^32^. Also, the antiproliferative activity against MCF-7 cells and wound healing activities of silver nanoparticles synthesized from *C. scalpelliformis* were studied and have proven that *C. scalpelliformis* aqueous extract modulated the expression of apoptotic genes^33^. Compared with the previous records of other *Caulerpa* species, fractionated polyphenols of *C. racemosa* suggest a potential antiproliferative activity with lower IC_50_ values.

As per previous studies, several bioactive molecules from *C. racemosa* are proven to indicate anticancer activity. Two such compounds are caulerpin and racemosin C, where caulerpin exhibited in vitro antitumour, antimicrobial, antinociceptive, anti-inflammatory activities and multixenobiotic resistance in (MXR) pump inhibitor^32^. The second isolated compound, racemosin C^34^, displayed PTP1B (protein tyrosine phosphatase 1B), a negative signal for insulin and leptin signalling inhibitory activity, and inhibition of PTP1B resulted in increased action of insulin and leptin^35^.

Breast cancer is common cancer diagnosed among women, and the action is mediated mainly via estrogen action and the presence of two estrogen receptors. Especially the presence of estrogen receptor alpha (Erα) is important for prognosis and a better response to hormonal therapy^36^. Therefore, we selected the Erα for the docking studies since the alpha receptor is important for developing the mammary glands and controlling nuclear DNA transcription^37^.

## Conclusion

The current study, backed with several scientific evidence, suggests the excellent use of marine algae- *C. racemosa* as a precursor for novel therapeutic approaches. Molecular in silico screening against estrogen receptor (ER) protein with caulerpin and the standard drug, mitoxantrone, revealed lower binding of − 11.18 kcal/mol compared to the standard drug (− 8.45 kcal/mol), giving the green light to the possibility of developing the CPE as an anticancer drug lead for breast cancer. Further, van der Waal and other attractive chargers were observed between the ER protein and the caulerpin. In contrast, the formation of three hydrogen bonds and two van der Waal interactions indicated the stability of the complex. However, the genus *Caulerpa* and its species have not been thoroughly investigated and therefore contain many more undiscovered compounds and therapeutic activities. Moreover, the studies have not adequately generalized to the in vivo models. Therefore, to formulate a novel drug, the genus *Caulerpa* and the species *C. racemosa* can provide dual benefits by being a more robust alternative food with therapeutic importance.

## Methods

### Chemical and reagents

The types of equipment used in this study were a gas chromatography-mass spectrometry (GC/MS; Spectra lab Agilent‐7890, Markham, ON, Canada) instrument, an Ultrasound sonicator (Elma D-78224 Singen/htw, Germany), a rotary evaporator (Buchi rotovapor, R‐124 digital, New Castle, DE, USA), Elisa microplate reader (BioBase EL-10A, China). All organic solvents and chemicals used during the study were of analytical grade. Water, when used, was distilled using distillation apparatus.

### Plant material

Samples of *Caulerpa racemosa* were collected from Hikkaduwa (6° 8′ 22.0848ʺ N and 80° 6′ 22.6260ʺ E) with permission obtained from the Department of wildlife conservation (No: WL/3/2/8/19). Upon collection of samples in January 2019, authentication was done by Dr Kalpa Samarakoon, a specialist in algal identification. Sample preparation was carried out as per. Initially, samples were cleaned thoroughly to remove the attached debris. Subsequently, they were lyophilized, ground into a fine powder, weighed and stored at − 20 °C until further use.

### Extraction of polyphenols

The powdered algae (100 g) were depigmented and defatted with acetone and n-hexane. The powder was then extracted thrice with 70% methanol using ultrasound-assisted extraction (at 25 °C for 90 min) and filtered to obtain the crude methanolic polyphenol extract (CPE). The resulting portion was obtained with an evaporation temperature of 40 °C and sequentially partitioned with equal volumes of n-hexane, chloroform, ethyl acetate, and distilled water.

### Total phenolic (TPC) and flavonoid content (TFC)

The total phenolic content of CPE was evaluated using the standard Folin Ciocalteu reagent^38^. A 20 µL of an aliquot of the dried residue of CPE and a standard solution of gallic acid dissolved in distilled water (0.03125, 0.0625, 0.125, 0.25, 0.5 mg/ml) diluted Folin Ciocalteu reagent and Na_2_CO_3_ (7.5%, 80 µL) in a microplate. After two hours of dark incubation at room temperature, the absorbance was 600 nm. TPC was expressed as mg gallic acid equivalents per gram of dried extract (mg GAE/ g).

The total flavonoid content was measured by the aluminum chloride colourimetric assay^39^. A 500 µL of crude extract (CPE), standard solution of quercetin dissolved in distilled water (0.03125, 0.0625, 0.125, 0.25, 0.5 mg/ml) was added to 500 µL of 2% (m/v) methanolic AlCl_3_.6 H_2_O. The absorbance was taken 15 min later at 430 nm. The results were expressed in milligrams compared with standard Quercetin equivalent per gram of dry weight treated in the same conditions.

### Antioxidant activity

The antioxidant activity of the crude polyphenolic extract (CPE) was examined using DPPH (2,2-diphenyl-1-picryl-hydrazyl-hydrate) and FRAP (ferric reducing antioxidant power) assays.

#### DPPH scavenging activity

For the DPPH assay, a DPPH solution in methanol and a stock solution of crude extract (800 μg/ml) with its dilutions were made. A hundred microliters of two-fold dilutions (12.5, 25, 50, 100, 200, 400) μg/ml of CPE were mixed with the same volume of DPPH solution in a 96-well microplate. After 30 min of dark incubation at room temperature, the absorbance was taken at 517 nm using a microplate reader. Ascorbic acid was used as the positive control (standard). The blank was 200 μl methanol, while the negative control was 100 μl of methanol mixed with 100 μl DPPH solution^40^.

#### Ion chelating ability

The FRAP reagent was freshly prepared using 10 mM of 2,4,6-Tris (2-pyridyl)-1,3,5-triazine (TPTZ) (dissolved in 40 mM of HCl), 20 mM of FeCl_3_ in water and 300 mM of acetate buffer (pH 3.6) in the ratio of 1:1:10. After loading the samples with FRAP reagent in microplates, they were incubated for 90 min (at 37 °C) before recording the absorbance at 600 nm. Vitamin C (l-ascorbic acid) was used as the antioxidant standard. The sample absorbance was compared to a standard curve of FeSO_4_. The FRAP values were expressed as Ferrous Equivalent (FE), the concentration of extract or chemical that gives the same absorbance as one mmol ferrous ion^40^.

### Gas chromatography/mass spectrometry (GC/MS) analysis

Since the ethyl acetate fraction of *Caulerpa racemosa* exhibited strong hypoglycemic activities, it was subjected to gas chromatography/mass spectrometry (GC–MS). The analysis was carried out using a Hewlett Packard Gas Chromatograph (Spectra lab Agilent-7890—Canada) equipped with a triple-axis detector and Hewlett Packard series injector (7683 B), keeping the MS transfer line temperature at 250 °C. The GC was fitted with a fused silica capillary column- HP-5MS (30 × 0.25 mm) of the film thickness of 1.0 μm. The oven temperature was initially maintained at 50 °C for 5 min and raised from 50 to 250 °C at a rate of 3 °C/min, employing helium gas (99.999%) at a constant speed of 22 cm/s. The extract, weighted 1.0 micron (1 mg dissolved in 1 ml of ethyl acetate), was injected at a split ratio of 1:30^41^. Similarly, MS analysis was carried out on Agilent Technology Network Mass Spectrometer (model 5975 series) coupled with the Hewlett Packard Gas Chromatograph (Model 7890 series) equipped with the NIST08 library software database. Mass spectra were taken at 70 eV/200 °C, with a scanning rate of 1 scan/s. The mass spectrums of individual unknown compounds were compared to the known compounds stored in the NIST08 library software database.

### Alpha-amylase inhibition assay

A 40 µL of test samples (fractions of algae) prepared in DMSO (concentration: 31.25–1000) µg/ml was reconstituted in 160 µL of Phosphate buffer (100 mM, pH = 6.9) and incubated for 5 min with 200 µL of yeast alpha-amylase (4 U/ml) prepared in ice-cold distilled water. Upon the addition of 400 µL soluble starch (0.5% w/v in 20 mM Phosphate buffer pH = 6.9) and 3 min of incubation, 400 µL of DNS was added, and closed tubes were placed in a hot water bath (80–90 °C for 10 min) to develop colour and were let to cool. Finally, 50 µL of the mixture was diluted with 175 µL of distilled water in a microplate and absorbance was taken at 504 nm^41^.

### Alpha-glucosidase inhibition assay

A 20 µL of test samples (fractions of algae) prepared in DMSO (31.25–1000) µg/ml were reconstituted in 100 µL of Phosphate buffer (100 mM, pH = 6.8) and were incubated for 5 min with 50 µL of yeast alpha-glucosidase (0.76 U/ml) prepared in the same buffer. After, 50 µL of 5 mM p-NPG prepared in the same buffer was added and incubated for 5 min at 37 °C. The absorbance was measured at 405 nm^3^. The positive control was acarbose, while the negative control included all the solvents with DMSO.

### Antiproliferative activity

The Institutional Review Board (IRB) Ministry of National Guard Health Affairs, Kingdom of Saudi Arabia (RC13/267) approved the study, where antiproliferative studies were conducted. Human breast cancer (MCF-7), breast epithelial cancer (MDA-MB-231), colorectal cancer (HCT-8), hepatic cancer (Hep G2), leukemia (erythroleukemia: KG-1a; lymphoblastic: K-562; acute myeloid leukemia: HL-60), and normal breast epithelial (MCF-10A) cell lines were purchased from ATCC, USA. The KAIMRC 1 cell line and primary blood monocular cells (PBMC) were isolated and established at King Abdullah International Medical Research Center). Mitoxantrone was used as the positive control.

To determine the effect of the algae fractions on the proliferation of the non-adherent cells, the CellTiter-Glo assay (Promega) was used according to the manufacturer’s recommendations. The Luminescence was measured (Envision plate reader; Perkin Elmer), normalized to averaged DMSO controls, and expressed as a relative percentage. The cells were seeded in 96-well plates with a growth medium and various extract concentrations, and each fraction ranged from 0 to 250 μM. Cells were incubated at 37 °C for 24 h, and CellTiter-Glo reagent was added to each well, mixed for 2 min, and luminescence was determined using an Envision plate reader (Perkin Elmer). Half-maximal inhibitory concentration IC_50_ values (μM) were calculated for each^42^.

The above procedure was followed for the MTT assay. Instead of the CellTiter-Glo reagent, the MTT reagent (Sigma) was added to evaluate the algae fractions’ effect on the adherent cells' proliferation. Absorbance was measured using the Spectra max spectrophotometer (Invitrogen), normalized to averaged DMSO controls, and expressed as a relative percentage^43^.

### Selectivity indices

To determine the specificity of a drug against a respective cancer cell, a selectivity index was calculated^29^. It can be determined by the ratio of IC_50_ of a standard cell line and the separate cancerous cell line (SI = IC_50_ for normal cell line / IC_50_ for cancerous cell line).

### Molecular docking

#### Protein structure preparation for docking studies

The structure of the selected proteins was accessed using the protein data bank (PDB) server and downloaded using the accession number 5nn 8 for human α-glucosidase and 3os8 for the Estrogen receptor protein. For α-glucosidase protein, chain A was selected, and for Estrogen receptor alpha protein, both A and B chains were selected. For both proteins, heteroatoms were removed. To prepare the proteins for docking, studies were done by deleting all water molecules and afterwards, polar hydrogen atoms were added. Kollman charges were also added in preparing the proteins for the docking studies. The protein was saved in PDBQT format for further analysis.

#### Preparation of the ligand for docking

The ligand of selection was an isolated compound, caulerpin, and this was used in further analysis in molecular docking studies. The ligand structure was downloaded using the PubChem database. The structure was energy minimized using the Chem3D (version 12.0), and the resulting structure was saved in PDB format. RasWin software was used to visualize the ligand structure. The ligand was prepared for docking by adding Gasteiger charges and saved in PDBQT format^44^.

### Statistical analysis

Statistical analysis was conducted using Minitab 17 software. Correlations were performed using “R” statistical software, while antiproliferative activity was determined using GraphPad Prism (version 9) software, and the half-maximal inhibitory concentration (IC_50_) was determined. Three replicates were performed to finalize the results of each experiment. The mean and the standard deviation were calculated. One-way ANOVA was used to determine the significant difference between each sample. P < 0.05 was considered significant.

### Ethical approval and informed consent

The authors declare that all methods were carried out following relevant guidelines and regulations. The authors confirm that all experimental protocols were approved by the Review Board (IRB) Ministry of National Guard Health Affairs, Kingdom of Saudi Arabia (RC13/267) .

## Data Availability

The data are included in the manuscript.

## Abbreviations

CPE: Crude polyphenolic extract
DPPH: 2,2-Diphenyl-1-picrylhydrazyl
ER: Estrogen receptor
FDA: Food and drug Administration
FRAP: Ferric reducing antioxidant power
GAE/g: Gallic acid equivalent in milligrams per gram dry plant extract
GC/MS: Gas chromatography/mass spectrometry
MTT: 3-(4,5-Dimethylthiazol-2-yl)-2,5-diphenyl-2H-tetrazolium bromide
MXR: Multixenobiotic resistance
PTP1B: Protein tyrosine phosphatase 1B
QC/g: Quercetin equivalent in milligrams per gram dry plant extract
TFC: Total flavanoid content
TPC: Total phenolic content

## Material availability

For the collection of plants, all relevant permits or permissions have been obtained from the Department of wildlife conservation (No: WL/3/2/8/19). The study also complies with local and national regulations.
