# Author Correction: Integration of in vitro and in-silico analysis of *Caulerpa racemosa* against antioxidant, antidiabetic, and anticancer activities

**DOI:** 10.1038/s41598-023-30967-4

**Published:** 2023-03-09

**Authors:** Indeewarie H. Dissanayake, Upeka Bandaranayake, Lakshika R. Keerthirathna, Chamalika. Manawadu, Rajitha M. Silva, Boudjelal Mohamed, Rizwan Ali, Dinithi C. Peiris

**Affiliations:** 1 Department of Zoology, University of Sri Jayewardenepura, Nugegoda, 10250 Sri Lanka; 2 Department of Statistics, University of Sri Jayewardenepura, Nugegoda, 10250 Sri Lanka; 3 Light and Electron Microscopy Unit, Medical Core Facility and Research Platforms, King Abdullah International Medical Research Centre, National Guard Health Affairs, Mail Code 1515, P.O. Box 3660, Riyadh, 11481 Saudi Arabia; 4 King Abdullah International Medical Research Center (KAIMRC), Medical Research Core Facility and Platforms (MRCFP), King Saud bin Abdulaziz University for Health Sciences (KSAU-HS), Ministry of National Guard Health Affairs (MNGHA), Riyadh, 11481 Saudi Arabia; 5 Department of Zoology/Genetics and Molecular Biology Unit (Centre for Biotechnology, University of Sri Jayewardenepura, Nugegoda, 10250 Sri Lanka

Correction to: *Scientific Reports* https://doi.org/10.1038/s41598-022-24021-y, published online 02 December 2022

The original version of this Article contained an error in the spelling of the author Rizwan Ali which was incorrectly given as Ali Rizwan.

In addition, Rizwan Ali was incorrectly affiliated with ‘Light and Electron Microscopy Unit, Medical Core Facility and Research Platforms, King Abdullah International Medical Research Centre, National Guard Health Affairs, Mail Code 1515, P.O. Box 3660, Riyadh 11481, Saudi Arabia’. The correct affiliation is listed below.

King Abdullah International Medical Research Center (KAIMRC), Medical Research Core Facility and Platforms (MRCFP), King Saud bin Abdulaziz University for Health Sciences (KSAU-HS), Ministry of National Guard Health Affairs (MNGHA), Riyadh 11481, Saudi Arabia.

The original Article has been corrected.

